# Moderate densification and fertilization enhance water use efficiency in foxtail millet by optimizing water consumption partitioning

**DOI:** 10.3389/fpls.2026.1758305

**Published:** 2026-02-18

**Authors:** Jinhuan Zheng, Yawei Li, Tianpeng Liu, Lei Zhang, Jihong He, Kongjun Dong, Ruiyu Ren, Yiyou Chen, Tianyu Yang

**Affiliations:** 1 Crop Research Institute, Gansu Academy of Agricultural Sciences, Lanzhou, China; 2 College of Life Science and Technology, Gansu Agricultural University, Lanzhou, China; 3 Gansu Academy of Agricultural Sciences, Lanzhou, China

**Keywords:** evapotranspiration, foxtail millet, grain yield, organic fertilizer, soil evaporation

## Abstract

**Introduction:**

Moderate densification and fertilization are widely used in agricultural practice due to their advantages in improving crop population structure, but it is currently unclear whether the organic combination of the two can optimize crop water consumption characteristics and improve water use efficiency (WUE).

**Methods:**

A split-plot design, which contained the main plot setting three fertilization levels: N1 (organic fertilizer only), N2 (inorganic fertilizer only), and N0 (no fertilizer) and the sub-plots setting three densification levels: D1 (200,000 plants hm⁻²), D2 (400,000 plants hm⁻²), and D3 (600,000 plants hm⁻²), was employed to investigate the combined effects of the two factors on the water use characteristics in foxtail millet.

**Results:**

The results showed that N2D2 significantly increased the leaf area index (LAI), aboveground biomass (AB) and root biomass (RB) of foxtail millet, and the grain yield was increased by 0.75% − 38.62% compared with other treatments. Meanwhile, the N2D2 treatment significantly reduced soil evaporation (E), increased plant transpiration (T), and raised the transpiration/evapotranspiration ratio to 61.44% − 62.03%. The soil water storage (SWS) capacity remained at a relatively high level in the 0−100 cm soil layer. Ultimately, the WUE of this treatment was significantly increased by 3.41% − 35.64%, and the water consumption structure was optimal. The structural equation model further revealed that the increase in WUE was mainly attributed to the interaction effect of fertilization and density, which promoted root biomass (RB) increase by optimizing SWS in the 0−40 cm soil layer, thereby influencing AB to positively regulate WUE.

**Discussion:**

In conclusion, under the condition of chemical nitrogen fertilizer application, moderate densification (400,000 plants hm⁻²) optimizes the population structure and water consumption patterns, synergistically enhancing both crop yield and water use efficiency. This approach represents an effective agronomic practice for achieving high−yield and water−saving cultivation of foxtail millet in arid regions.

## 1 Introduction

Against the backdrop of escalating global climate change and increasing agricultural water scarcity, improving water use efficiency (WUE) in dryland farming systems has become a critical scientific issue for ensuring regional food security and sustainable agricultural development (Wu et al., 2025; Yang et al., 2025). Foxtail millet (*Setaria italica*), as a major drought-tolerant cereal crop in northern China, exhibits strong adaptability to water-limited environments (Taylor et al., 2010). Its WUE not only influences yield stability but also affects the optimization of regional water resources and the livelihoods of local farmers (Zhai et al., 2022). However, the actual WEP in major foxtail millet production areas remains generally low, largely due to prolonged dependence on traditional extensive cultivation practices (Bennetzen et al., 2012; Li et al., 2024). This results in a significant gap from its theoretical potential as a C4 crop. This gap stems primarily from two interrelated agronomic constraints: first, imbalanced nitrogen management often disrupts crop water-use strategies, where excessive application despite stimulating growth frequently induces luxury transpiration and consequently lowers WUE (Fernández-Ortega et al., 2023; Zhuang et al., 2024); second, suboptimal planting densities lead to inefficient canopy architecture, which not only compromises light and heat utilization but also exacerbates water competition among plants (Gao et al., 2021; Yu et al., 2025).

The full plastic film mulching on double ridges and planting in furrows (FPMDR) system has been widely adopted as an effective water-conserving practice in dryland agriculture, primarily by suppressing soil evaporation and enhancing rainfall infiltration to create a stable root-zone moisture environment (Li et al., 2023; Liu et al., 2023a). Nevertheless, the water-saving potential of this system depends critically on the synchronized management of fertilization and crop population structure (Wang et al., 2020). Therefore, advancing from single-factor water conservation to the integrated optimization of water, fertilizer, and planting density is essential to unlock the system’s full WUE-enhancement potential (Cao et al., 2024). Fertilization strategies play a decisive role in regulating crop growth and WUE. Chemical and organic fertilizers differ fundamentally in their mechanisms of action: chemical nitrogen tends to promote rapid canopy development and photosynthetic activity, yet may encourage shallow root systems that limit access to deeper soil moisture (Wang et al., 2024a; Ren et al., 2025); organic amendments enhance soil structure and water-holding capacity but may not meet the crop’s peak nutrient demand during critical growth stages (Ren et al., 2022; Zhang et al., 2025). Concurrently, planting density directly shapes canopy architecture and resource competition. Both excessively high and low densities can reduce WUE: the former by accelerating soil water depletion and inducing premature senescence, the latter by failing to fully utilize available radiation and soil water (Koffi Djaman et al., 2022). Critically, fertilization and planting density interact in shaping crop water use. Under optimized nutrient supply, a moderate increase in planting density may improve canopy light interception and adjust the proportion of transpiration relative to evaporation, thereby enhancing WUE at the population level (Xiao et al., 2023; Jiao et al., 2023). However, this interaction is complex and modulated by genotype, soil moisture dynamics, and environmental conditions. Most existing studies have focused on the isolated effects of either fertilizer type or planting density, while the integrated effects and physiological mechanisms governing their combined impact on WUE remain insufficiently explained.

To address these research gaps, a field experiment was conducted at the Huining Experimental Station in Gansu Province from 2023 to 2024 under the FPMDR system. Using a split-plot design, this study aimed to test two key hypotheses: (1) that chemical nitrogen fertilizer would enhance WUE more effectively than organic or no fertilizer, but that excessively high planting density could negate this benefit by intensifying water competition; and (2) that a combination of moderate planting density with chemical nitrogen would optimize the water consumption structure by increasing productive transpiration while reducing non-productive evaporation, thereby synergistically improving both grain yield and WUE. Through this work, we seek to elucidate the agronomic and physiological mechanisms underlying WUE improvement and to provide a scientific basis for designing integrated cultivation strategies that support high-yield and water-efficient foxtail millet production in arid regions.

## 2 Materials and methods

### 2.1 Experimental site

**Figure 1:**
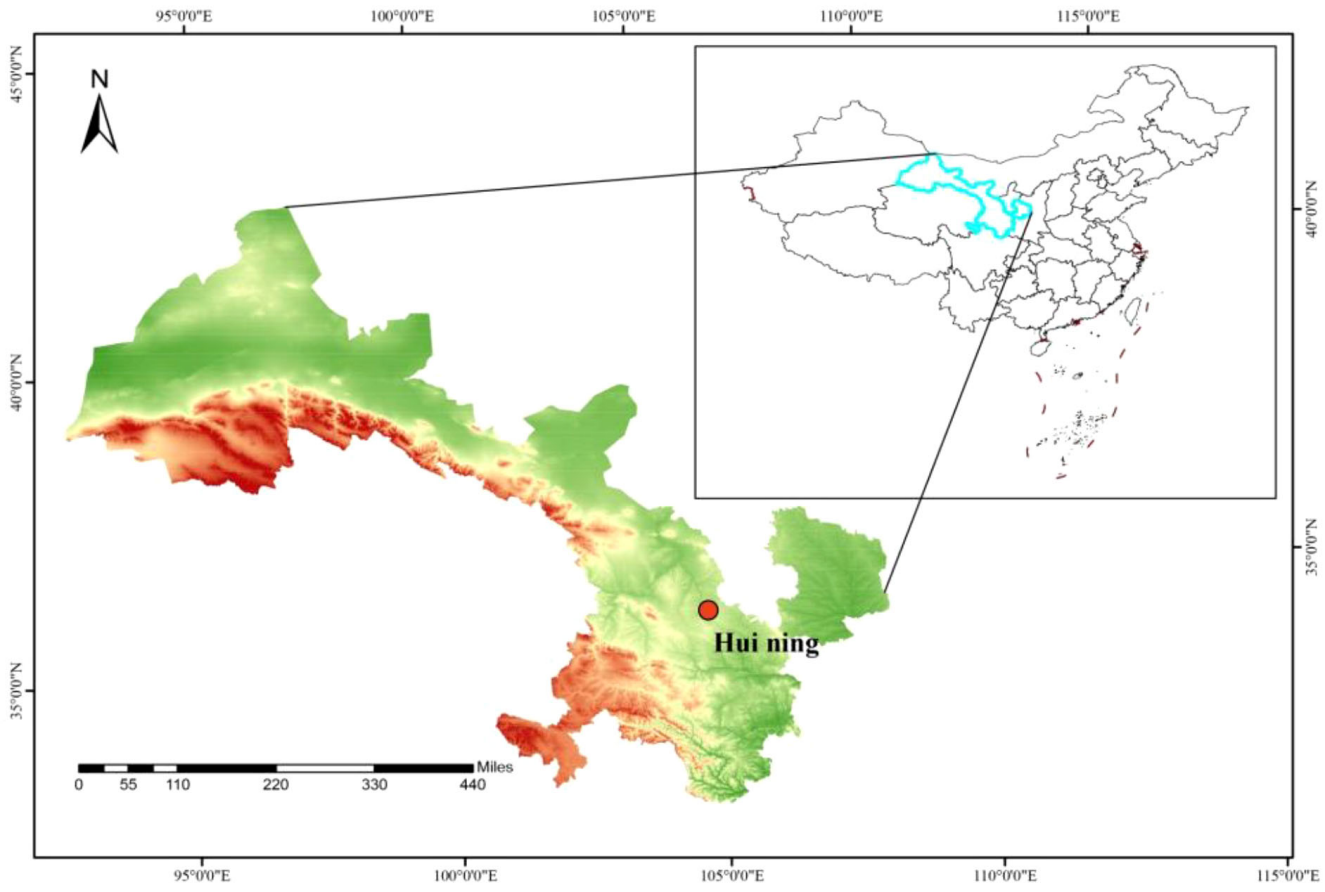
Location of the experimental area.

**Figure 2:**
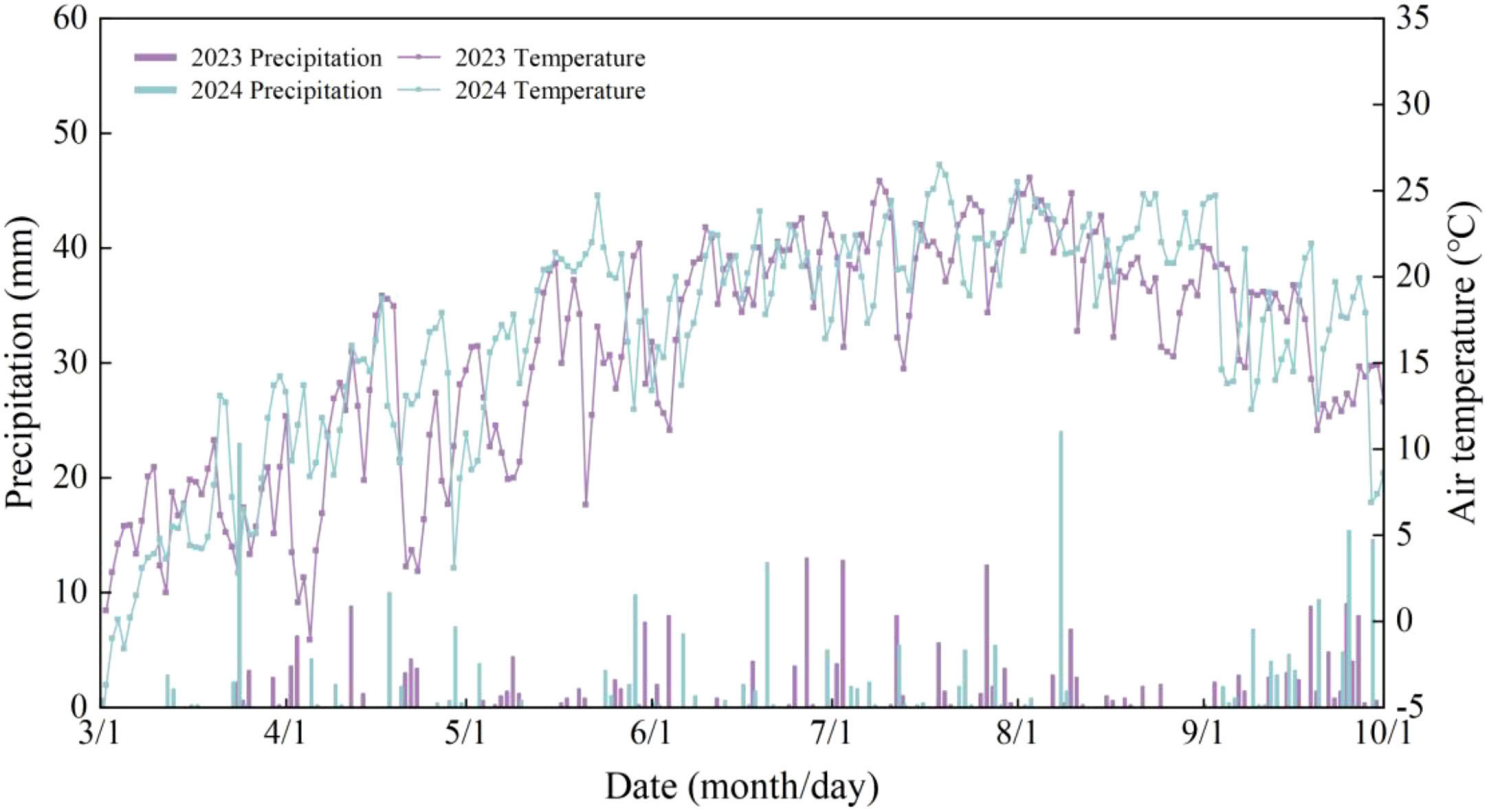
Average air temperatures and precipitation for 2023 and 2024.

The study was conducted at the Huining Experimental Station (35°40′N, 105°06′E; 1800 m altitude) of Gansu Academy of Agricultural Sciences in Gaoling Village, Zhongchuan Town, Huining County, Gansu Province (**Figure 1**). The region is characterized by a temperate semi-arid continental monsoon climate. The site has a mean annual temperature of 6.8 °C and a frost-free period of approximately 130 days, with mean annual precipitation of 360 mm and evaporation of 1800 mm. The experimental soil was loessal soil with: 12.2 g kg^−1^ organic matter, 0.83 g kg^−1^ total N, 44.6 mg kg^−1^ available N, 0.83 g kg^−1^ total P, 3.79 mg kg^−1^ available P, 20.6 g kg^−1^ total K, and 178 mg kg^−1^ available K. Average temperatures and precipitation for 2023 and 2024 are shown in **Figure 2**.

### 2.2 Experimental design

A split-plot field experiment was conducted under the complete plastic film mulching with double ridges and furrow sowing system. The main plots were assigned to three fertilization regimes: N1 (organic fertilizer only), N2 (inorganic fertilizer only), and N0 (no fertilizer). Subplots were allocated to three planting densities: D1 (200,000 plants hm⁻²), D2 (400,000 plants hm⁻²), and D3 (600,000 plants hm⁻²) (**Table 1**). The experiment comprised a total of nine treatments, each replicated three times in a randomized complete block design. The planting was performed by hand dibbling in holes spaced 20 cm apart within rows, with row spacing of 40 cm and a ridge width of 60 cm. Each subplot measured 5 m in length and 4 m in width, with a total area of 20 m². Sowing was performed in mid-May each year. For the fertilization treatments, nitrogen was applied basally before planting at a rate of 120 kg N hm⁻²: in N1, this was supplied by a commercial organic fertilizer containing 5.0% total nitrogen, 2.5% total phosphorus (P_2_O_5_), and 2.0% total potassium (K_2_O); in N2, nitrogen was supplied by urea (46% N). The organic fertilizer was evenly broadcast and incorporated into the top 0–20 cm soil layer during field preparation, while urea was band-applied next to the sowing holes at a depth of 5 cm. No additional topdressing was applied during the growing season. Two widely cultivated foxtail millet varieties, ‘Nianbocaign’ and ‘Hongniangu’, were tested concurrently under all treatment combinations.

**Table 1:** Summary of experimental treatments combining fertilization and planting density.

Fertilization treatment	Planting density treatment (plants hm⁻²)	Treatment code
N0 (No fertilizer)	200,000 (D1)	N0D1
N0 (No fertilizer)	400,000 (D2)	N0D2
N0 (No fertilizer)	600,000 (D3)	N0D3
N1 (Organic fertilizer only)	200,000 (D1)	N1D1
N1 (Organic fertilizer only)	400,000 (D2)	N1D2
N1 (Organic fertilizer only)	600,000 (D3)	N1D3
N2 (Inorganic fertilizer only)	200,000 (D1)	N2D1
N2 (Inorganic fertilizer only)	400,000 (D2)	N2D2
N2 (Inorganic fertilizer only)	600,000 (D3)	N2D3

### 2.3 Measurements and methods

#### 2.3.1 Soil water content

Soil water content was determined using the oven-drying method at 10–15 day intervals from sowing to harvest, with additional measurements conducted following significant rainfall events. Measurements were taken at depth increments of 0–20, 20–40, 40–70, and 70–110 cm within each plot using a soil auger. The gravimetrically determined soil water content (%) was subsequently converted to volumetric water content (mm) by incorporating measured soil bulk density values for each corresponding layer (Nie et al., 2023).

#### 2.3.2 Soil water storage

Soil water storage (SWS, mm) in the 0–100 cm layer was calculated before sowing and after harvest using (Yin et al., 2018):

(1) SWS=SD×h×10

where SD represents volumetric soil water content (cm³ cm⁻³) and h is soil depth increment (cm).

#### 2.3.3 Soil evaporation

Soil evaporation (E, mm) between crop rows was measured using mini-lysimeters constructed from PVC tubes (15 cm length, 11 cm inner diameter, 11.5 cm outer diameter), sealed at the bottom with waterproof tape (Plauborg, 1995). Installed in plot centers within 12 cm outer PVC sleeves, measurements were taken every 5–7 days using a 1 g precision balance (1 g weight loss = 0.1053 mm evaporation).

#### 2.3.4 Evapotranspiration

Total evapotranspiration (ET, mm) was calculated via water balance equation (Chai et al., 2014):

(2) $$ET=P_{e}+I+U-R-D_{W}-\Delta S$$

where P_e_ denotes effective precipitation (USDA-NRCS method) (Kuo et al., 2006), I irrigation amount, U capillary rise, R runoff, and Dw deep drainage. In arid regions, U, Dw and R were considered negligible (Yang et al., 2011; Yin et al., 2019).

#### 2.3.5 Transpiration

Transpiration (T, mm) was derived as:

(3) T=ET−E

where E is soil evaporation. Capillary rise (CE) was negligible in drylands (Lawrence et al., 2007).

#### 2.3.6 Aboveground biomass, root biomass and LAI

Leaf area dynamics were measured at seven key growth stages: seedling stage, jointing stage, booting stage, heading stage, flowering stage, grain-filling stage, and maturity stage. At physiological maturity, five representative plants per plot were sampled, oven-dried at 105 °C for 30 minutes (initial deactivation), and then dried at 80 °C to constant weight for dry matter determination. Leaf area was measured at each of the seven aforementioned stages using the punch method. Leaf area was determined via punch method:

(4) Leaf area=(Leaf mass×total disc area)/total disc leaf mass

(5) LAI=(Leaf area×plant density)/unit land area

#### 2.3.7 Grain yield and water use efficiency

Six central rows (15 hills/row) were harvested separately (**Table 2**). Grain yield (kg hm⁻2) was determined after natural drying. Water use efficiency (WUE, kg hm⁻2 mm⁻¹) was calculated as:

**Table 2:** Summary of key parameters measured, their sampling times and methods.

Parameter	Sampling time/growth stage	Measurement method
SWC	From sowing to harvest (every 10–15 days and after significant rainfall)	Oven-drying method (gravimetric), converted to volumetric water content using measured bulk density
SWS	Before sowing and after harvest	Calculated from soil water content in the 0–100 cm soil profile
E	Every 5–7 days during the growing season	Mini-lysimeters (PVC tubes, 11 cm inner diameter) weighed on a precision balance (1 g = 0.1053 mm evaporation)
LAI	Seven key stages	Punch method (leaf discs), calculated per unit ground area
AB, RB	At physiological maturity	Plant samples oven-dried to constant weight
GY	At physiological maturity	Harvest of six central rows, threshed, and naturally dried

(6) WUE=GY/ET×100

where GY is grain yield (kg hm⁻^2^) and ET represents seasonal total evapotranspiration (mm).

### 2.4 Statistical analysis

Data were analyzed using SPSS 17.0 (SPSS Inc., Chicago, IL, USA). Treatment differences were assessed by Duncan’s multiple range test at P<0.05. Data analysis revealed no significant inter-annual interaction effect on the experimental results. Structural equation modeling (SEM) was performed using AMOS 22.0 (IBM Corporation, Armonk, NY, USA) to examine causal relationships among variables.

## 3 Results

### 3.1 Differential responses of Yield and WUE to fertilization and density treatments

**Figure 3:**
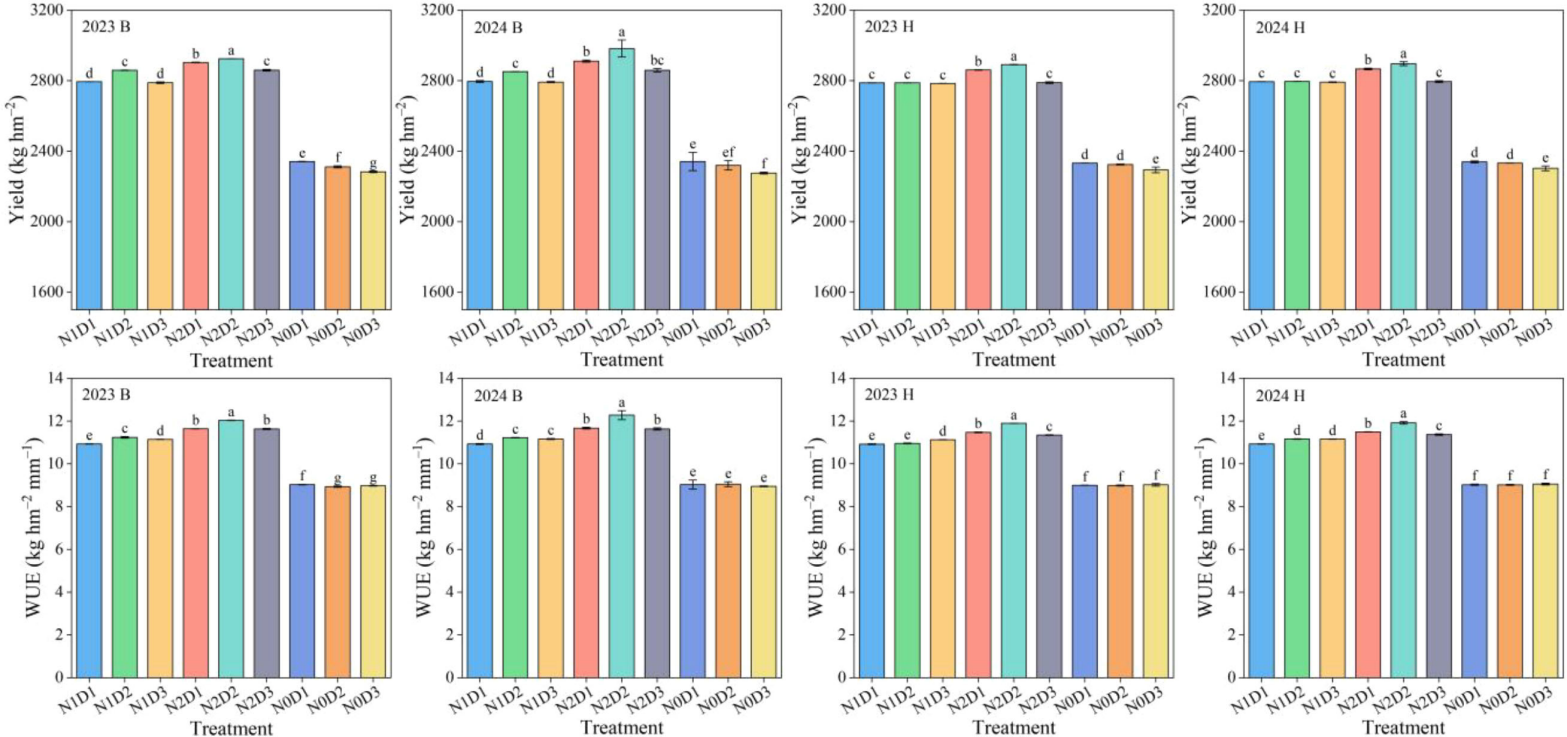
Foxtail millet yield and water use efficiency (WUE) under different treatments in 2023 and 2024. B and H represent the different millet varieties ‘Nianbocaign’ and ‘Hongniangu’, respectively. N, D, and N×D represent fertilization, density, and their interaction effects, respectively. Lowercase letters in the figure indicate significant differences between treatments within the same year (p< 0.05, n=3). Error bars indicate LSD (Least Significant Difference). ∗∗ indicates extremely significant differences (p< 0.01), ∗ indicates significant differences (p< 0.05), and ns indicates no significant differences.

Fertilization (N), density (D), and their interaction (N×D) significantly affected foxtail millet yield and water use efficiency (**Figure 3**). The water use efficiency (WUE) was calculated using Equation 6. For variety B, under different fertilization levels, the yield of N2 treatment increased by 3.05%−3.92% and 25.45%−26.43% compared to N1 and N0, respectively. Under different density levels, the yield of D2 treatment increased by 2.48%−3.09% and 3.87%−4.67% compared to D1 and D3, respectively. Under the interaction of fertilization and density (N×D), the yield of N2D2 treatment increased by 0.75%−2.47%, 2.29%−4.33%, 2.30%−4.60%, and 26.57%−38.62% compared to N2D1, N2D3, N1D2, and N0D2, respectively. Variety H also exhibited a similar pattern, with the yield of N2D2 treatment increasing by 1.04%−1.06%, 3.63%−3.66%, 3.61%−3.71%, and 24.20%−24.39% compared to N2D1, N2D3, N1D2, and N0D2, respectively. The N×D effect also significantly influenced millet WUE (**Figure 3**). For variety B, under N×D, the WUE of N2D2 treatment increased by 3.41%−5.21%, 7.09%−9.38%, and 34.69%−35.64% compared to N2D1, N1D2, and N0D2, respectively. For variety H, under N×D, the WUE of N2D2 treatment increased by 3.62%−3.81%, 6.77%−8.56%, and 32.15%−32.37% compared to N2D1, N1D2, and N0D2, respectively. In summary, the N2D2 treatment was beneficial for improving millet yield and WUE.

### 3.2 Differential responses of soil water storage to fertilization and density treatments

**Figure 4:**
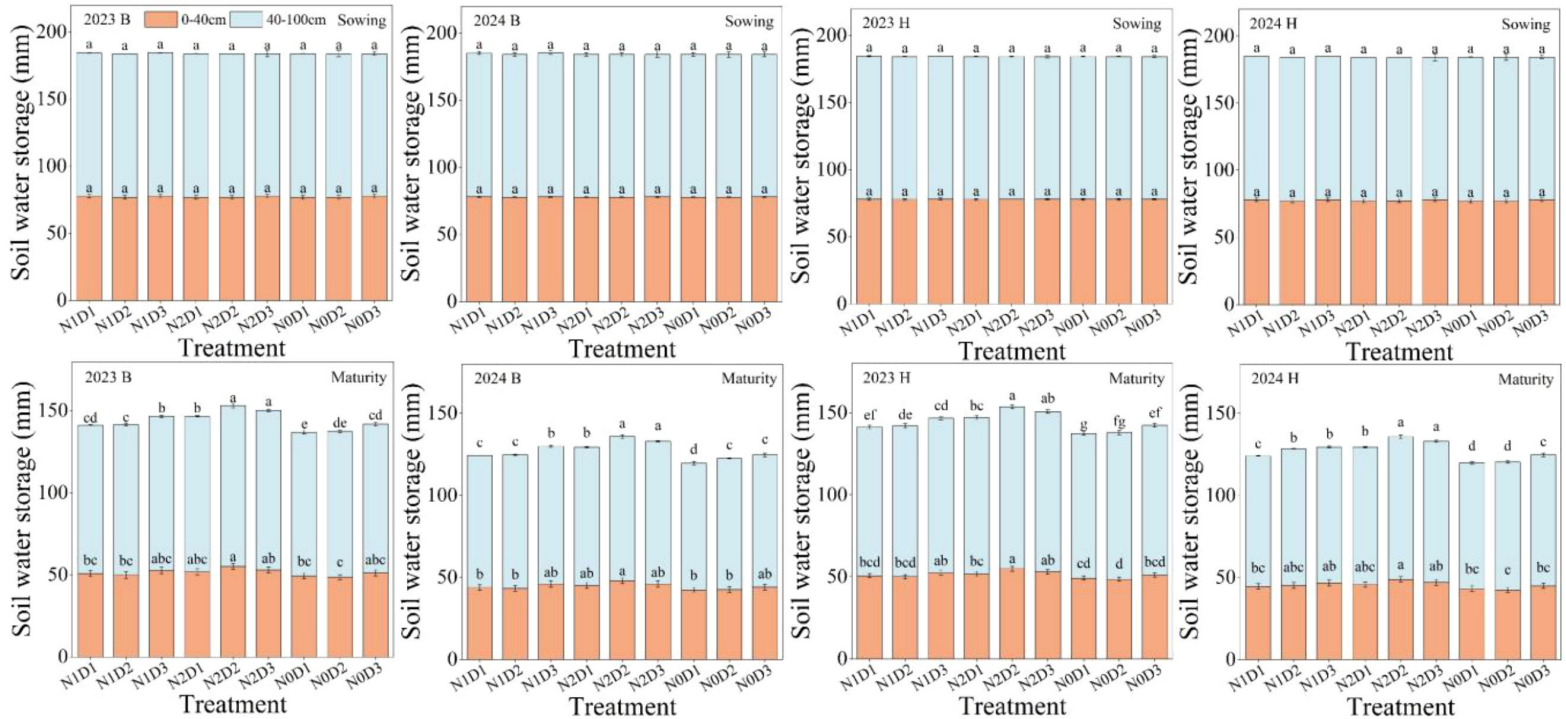
Soil water storage at sowing and maturity in the 0–40 and 40–100 cm soil layers under different treatments in 2023 and 2024. B and H represent the different millet varieties ‘Nianbocaign’ and ‘Hongniangu’, respectively. Lowercase letters in the figure indicate significant differences between treatments within the same year (p< 0.05, n=3). Error bars indicate LSD (Least Significant Difference).

There were no significant differences in SWS between the 0–40 cm and 40–100 cm soil layers under different treatments at the sowing stage. The soil water storage (SWS, mm) was calculated using Equation 1. However, at the maturity stage, N, D, and N×D significantly affected SWS in both the 0–40 cm and 40–100 cm soil layers (**Figure 4**). For variety B, under different fertilization levels in the 0−40cm soil layer, the SWS of N2 treatment increased by 4.64%−4.83% and 7.77%−8.25% respectively compared to N1 and N0. At different density levels, the SWS of D2 treatment increased by 2.21%−2.98% compared to D1 and decreased by 0.71%−1.05% compared to D3. At N×D, the SWS treated with N2D2 increased by 10.32%−10.69% and 12.71%−13.57% respectively compared to N1D2 and N0D2. In the 40−100cm soil layer, for different fertilization levels, the SWS of N2 treatment increased by 4.92%−5.31% and 8.35%−8.74% respectively compared to N1 and N0. At different density levels, the SWS of D2 treatment increased by 2.52%−3.37% compared to D1 and decreased by 0.41%−0.73% compared to D3. At N×D, the SWS treated with N2D2 increased by 3.25%−4.08%, 6.92%−7.86%, and 9.73%−10.15% respectively compared to N2D1, N1D2, and N0D2. Variety H also maintains a similar pattern of change. Under the N×D soil layer of 0−40cm, the SWS treated with N2D2 increased by 7.86%−9.98% and 13.28%−15.10% respectively compared to N1D2 and N0D2. In the 40−100cm soil layer under N×D, the SWS of N2D2 treatment increased by 3.33%−3.79%, 4.55%−6.97%, and 10.27%−11.63% compared to N2D1, N1D2, and N0D2, respectively. Overall, N2D2 treatment can maintain a high soil water storage capacity.

### 3.3 The effects of fertilization and density treatments on E, T and ET

N, D, and N×D significantly affected E and ET (**Table 3**). The total evapotranspiration (ET, mm) was calculated using the water balance equation (Equation 2). For variety B, at the fertilization level, the E and ET of N2 treatment decreased by 8.33%−8.54% and 2.83%−2.86%, 13.68%−13.71% and 4.05%−4.35%, respectively, compared to N1 and N0. At different density levels, the E and ET of D2 treatment decreased by 1.44%−2.51% and 1.41%−1.69%, 0.82%−0.95% and 0.16%−0.42%, respectively, compared to D1 and D3. At N×D, E and ET treated with N2D2 decreased by 11.46%−11.57% and 4.37%−4.48%, 16.28%−16.47% and 5.18%−6.02%, respectively, compared to N1D2 and N0D2. For variety H, under N×D, E and ET treated with N2D2 decreased by 11.40%−11.48% and 2.96%−4.47%, 16.45%−16.51% and 5.97%−6.03%, respectively, compared to N1D2 and N0D2. The varieties H, N, D, and N×D have a significant impact on T. The transpiration (T, mm) was derived as Equation 3. At the fertilization level, the T treated with N2 increased by 1.15%−1.85% and 2.75%−2.79% respectively compared to N1 and N0. At different density levels, the T of D2 treatment increased by 0.59%−1.63% and 0.33%−1.54% respectively compared to D1 and D3. At N×D, the T treated with N2D2 increased by 0.52%−3.15% and 1.88%−0.95% respectively compared to N1D2 and N0D2. For varieties B, N, and D, they have a significant impact on T, but N×D has no significant impact on T. In summary, N2D2 can reduce E and increase T to maintain a lower ET.

**Table 3:** Changes in soil evaporation, transpiration, and total evapotranspiration under different treatments in 2023 and 2024.

Year	Variety	Treatment	E (mm)	T (mm)	ET (mm)	Variety	E (mm)	T (mm)	ET (mm)
2023	B	N1D1	105.60 c	150.06 b	255.66 b	H	105.76 cd	149.58 ab	255.34 b
		N1D2	105.35 c	149.11 b	254.46 c		105.86 c	148.58 b	254.44 c
		N1D3	103.32 d	146.92 c	250.24 d		104.41 d	145.80 c	250.21 d
		N2D1	98.09 e	151.37 a	249.46 d		98.58 e	150.84 a	249.42 d
		N2D2	93.27 f	149.79 b	243.06 f		93.71 f	149.35 b	243.06 f
		N2D3	96.91 e	148.94 b	245.86 e		97.36 e	148.49 b	245.85 e
		N0D1	111.91 a	147.32 c	259.23 a		112.41 a	146.83 c	259.23 a
		N0D2	111.66 a	146.97 c	258.64 a		112.16 a	146.50 c	258.66 a
		N0D3	110.20 b	144.10 d	254.30 c		110.70 b	143.58 d	254.28 c
2024	B	N1D1	104.72 c	151.08 b	255.80 b	H	104.85 c	150.81 ab	255.66 b
		N1D2	104.34 c	149.75 d	254.09 c		104.65 c	145.81 d	250.46 d
		N1D3	102.06 d	148.14 e	250.20 d		102.59 d	147.66 c	250.24 d
		N2D1	96.76 e	152.73 a	249.49 e		97.37 e	152.09 a	249.46 e
		N2D2	92.27 g	150.72 bc	242.99 g		92.65 f	150.40 b	243.05 g
		N2D3	95.60 f	150.27 cd	245.87 f		96.22 e	149.64 b	245.86 f
		N0D1	110.51 a	148.67 e	259.18 a		111.22 a	147.98 c	259.20 a
		N0D2	110.21 a	146.06 f	256.27 b		110.97 a	147.63 c	258.60 a
		N0D3	109.01 b	145.29 g	254.30 c		109.55 b	144.77 d	254.32 c
N			∗∗	∗∗	∗∗		∗∗	∗∗	∗∗
D			∗∗	∗∗	∗∗		∗∗	∗∗	∗∗
N×D			∗∗	ns	∗∗		∗∗	∗∗	∗∗

E, T and ET represent soil evaporation, transpiration and evapotranspiration respectively. B and H represent the different millet varieties ‘Nianbocaign’ and ‘Hongniangu’, respectively. Lowercase letters in the figure indicate significant differences between treatments within the same year (p< 0.05, n=3). ∗∗ indicates significant differences (p< 0.001), and ns indicates no significant differences (p = 0.062).

### 3.4 Optimization of water consumption structure of foxtail millet by interaction between fertilization and density

**Figure 5:**
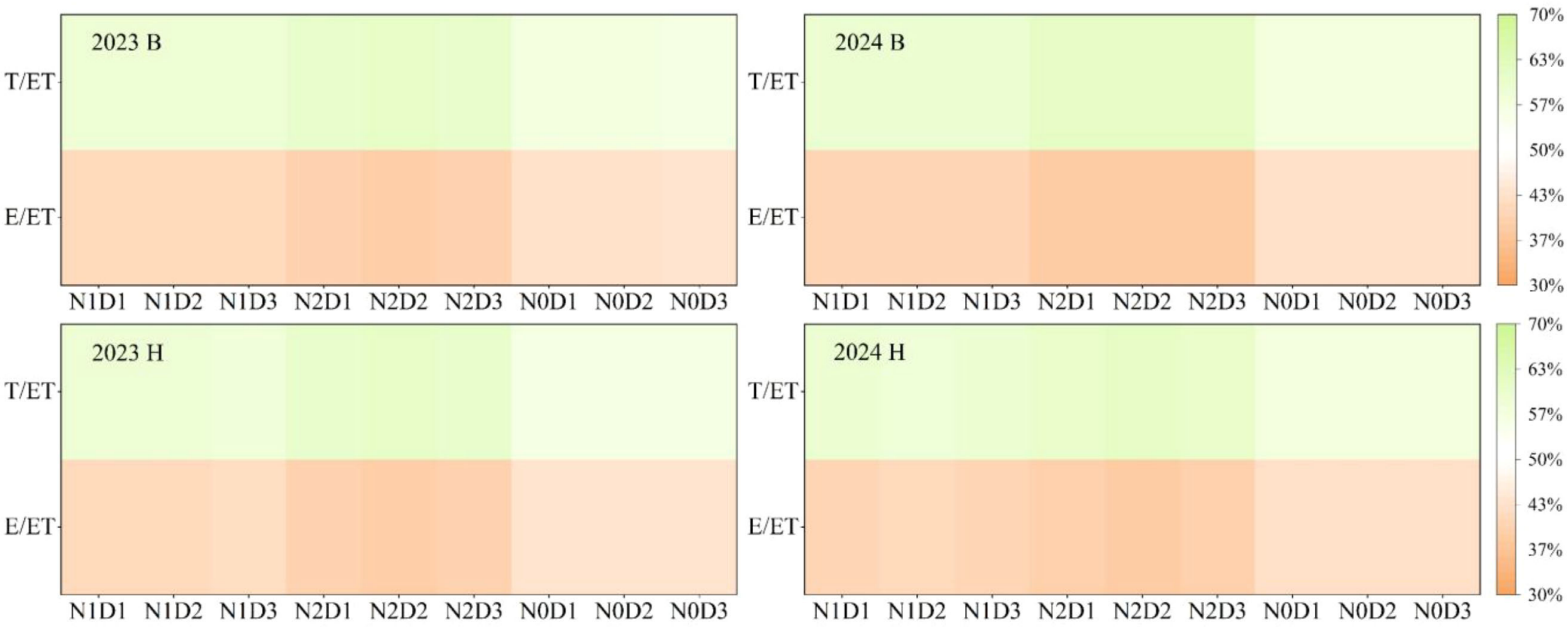
E/ET (soil evaporation/evapotranspiration) and T/ET (evapotranspiration/evapotranspiration) under different treatments in 2023 and 2024. B and H represent the different millet varieties ‘Nianbocaign’ and ‘Hongniangu’, respectively.

**Figure 5** showed the water consumption characteristics of foxtail millet under different treatments. The results indicated that for the two varieties B and H, the E/ET ratio was lowest under the N2D2 treatment, reaching 37.79%–38.56%. Meanwhile, the T/ET ratio was highest under the N2D2 treatment, reaching 61.44%–62.03%. It can be seen that the N2D2 treatment can optimize the water consumption characteristics of foxtail millet.

### 3.5 Differential responses of LAI to fertilization and density treatments

**Figure 6:**
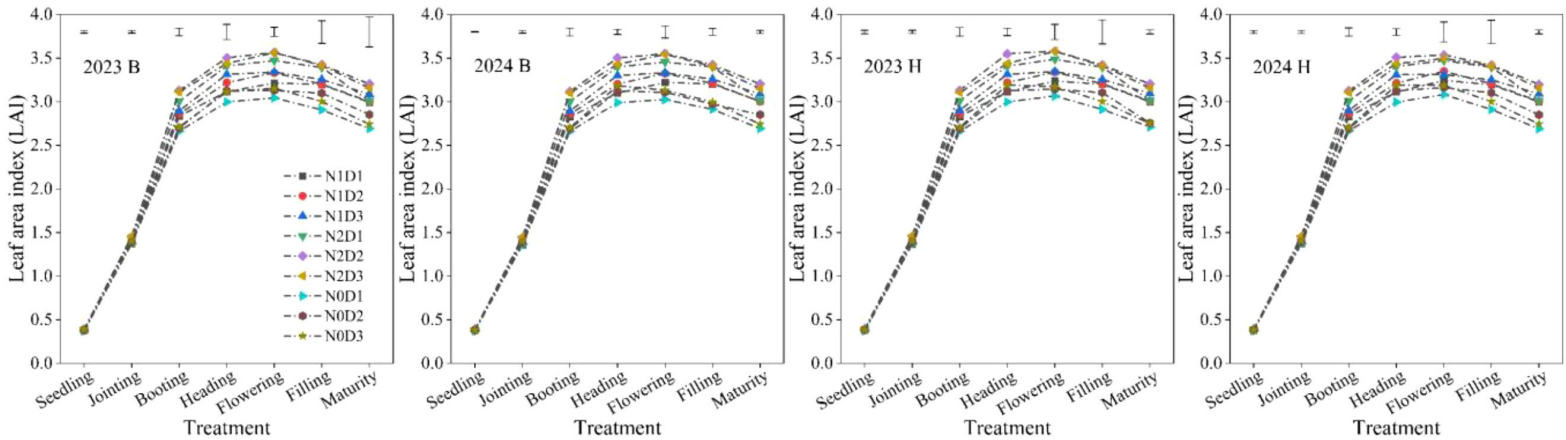
Leaf area index (LAI) of foxtail millet under different treatments in 2023 and 2024. B and H represent the different millet varieties ‘Nianbocaign’ and ‘Hongniangu’, respectively. Error bars indicate LSD (Least Significant Difference).

Under different treatments, the LAI of foxtail millet showed a trend of first increasing and then decreasing as the growth stage progressed (**Figure 6**). The leaf area index (LAl) was calculated using Equations 4, 5 and N×D had a significant effect on the LAI. During the flowering stage, LAI reached its highest value. For variety B, the N2D2 treatment increased by 6.86%−6.99% and 13.87%−14.25% respectively compared to the N1D2 and N0D2 treatments. For variety H, N2D2 treatment increased by 5.61%−7.24% and 11.84%−13.92% respectively compared to N1D2 and N0D2 treatments. Subsequently, the LAI of each treatment decreased until maturity. For variety B, the N2D2 treatment increased by 6.70%−6.73% and 12.27%−12.30% respectively compared to the N1D2 and N0D2 treatments. For variety H, N2D2 treatment increased by 6.49%−6.53% and 12.28%−16.26% respectively compared to N1D2 and N0D2 treatments.

### 3.6 Differential responses of aboveground biomass and root biomass to fertilization and density treatments

**Figure 7:**
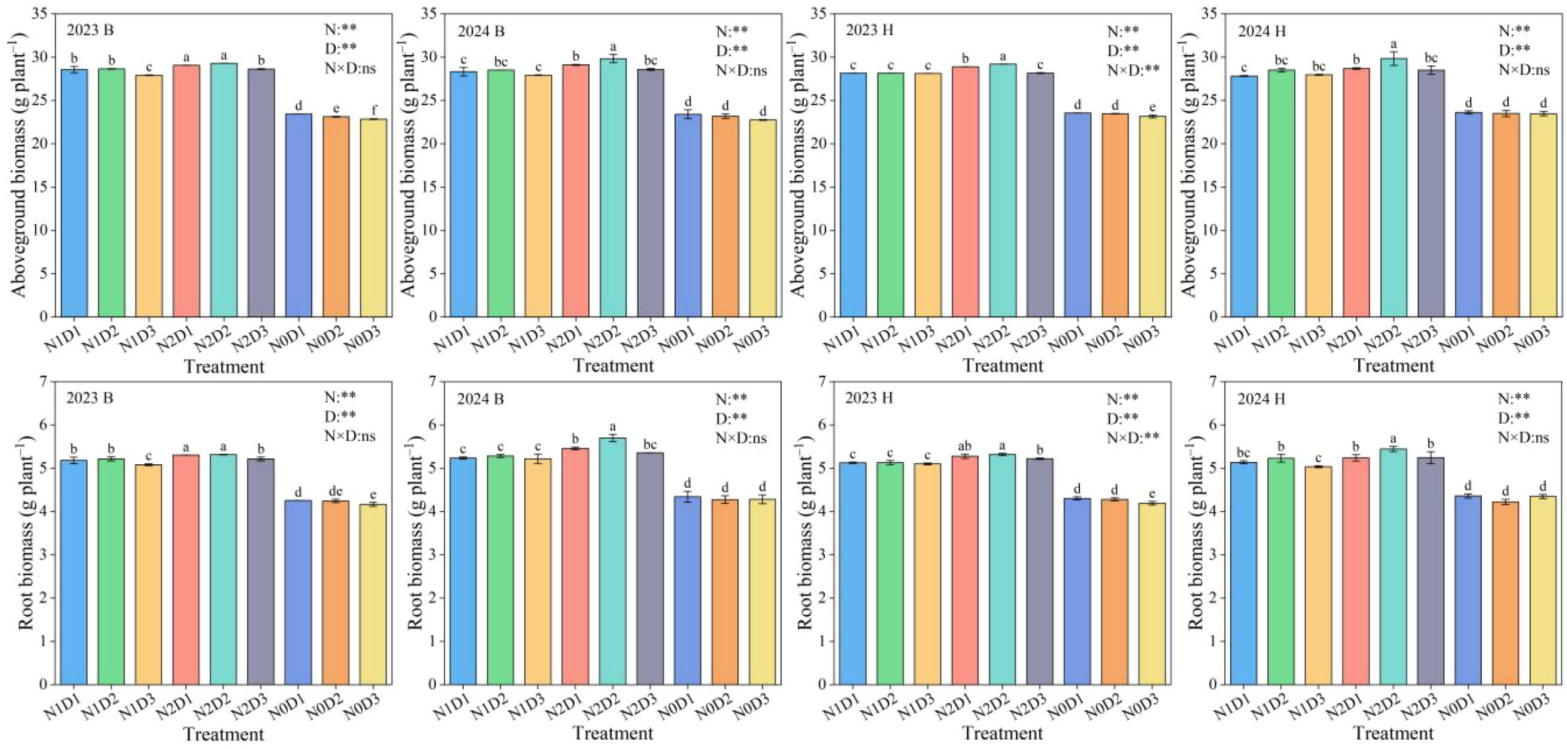
Aboveground biomass and root biomass of foxtail millet under different treatments in 2023 and 2024. B and H represent the different millet varieties ‘Nianbocaign’ and ‘Hongniangu’, respectively. N, D, and N×D represent fertilization, density, and their interaction effects, respectively. Lowercase letters in the figure indicate significant differences between treatments within the same year (p< 0.05, n=3). Error bars indicate LSD (Least Significant Difference). ∗∗ indicates extremely significant differences (p< 0.01), ∗ indicates significant differences (p< 0.05), and ns indicates no significant differences.

N, D, and N×D significantly affected the aboveground biomass and root biomass of foxtail millet (**Figure 7**). For variety B, under different fertilization levels, the aboveground biomass of foxtail millet treated with N2 increased by 2.18%−3.26% and 25.31%−26.19% respectively compared to N1 and N0. At different density levels, the aboveground biomass of foxtail millet treated with D2 increased by 0.09%−0.10% and 0.24%−0.32% respectively compared to D1 and D3. At N×D, the aboveground biomass of foxtail millet treated with N2D2 increased by 2.32%−4.43%, 2.24%−4.60%, and 26.62%−29.62%, respectively, compared to N2D3, N1D2, and N0D2. Variety H also maintained a similar pattern of change. Under N×D, the aboveground biomass of foxtail millet treated with N2D2 increased by 3.66%−4.65%, 3.71%−4.62%, and 24.39%−26.91%, respectively, compared to N2D3, N1D2, and N0D2. Similarly, for variety B, under N×D, the root biomass of foxtail millet treated with N2D2 increased by 1.91%–7.89% and 25.17%–33.28% compared to N1D2 and N0D2, respectively. For variety H, under N×D, the root biomass of foxtail millet treated with N2D2 increased by 3.63%–4.09% and 24.21%–28.97% compared to N1D2 and N0D2, respectively.

### 3.7 Correlation between WUE and various indicators

**Figure 8:**
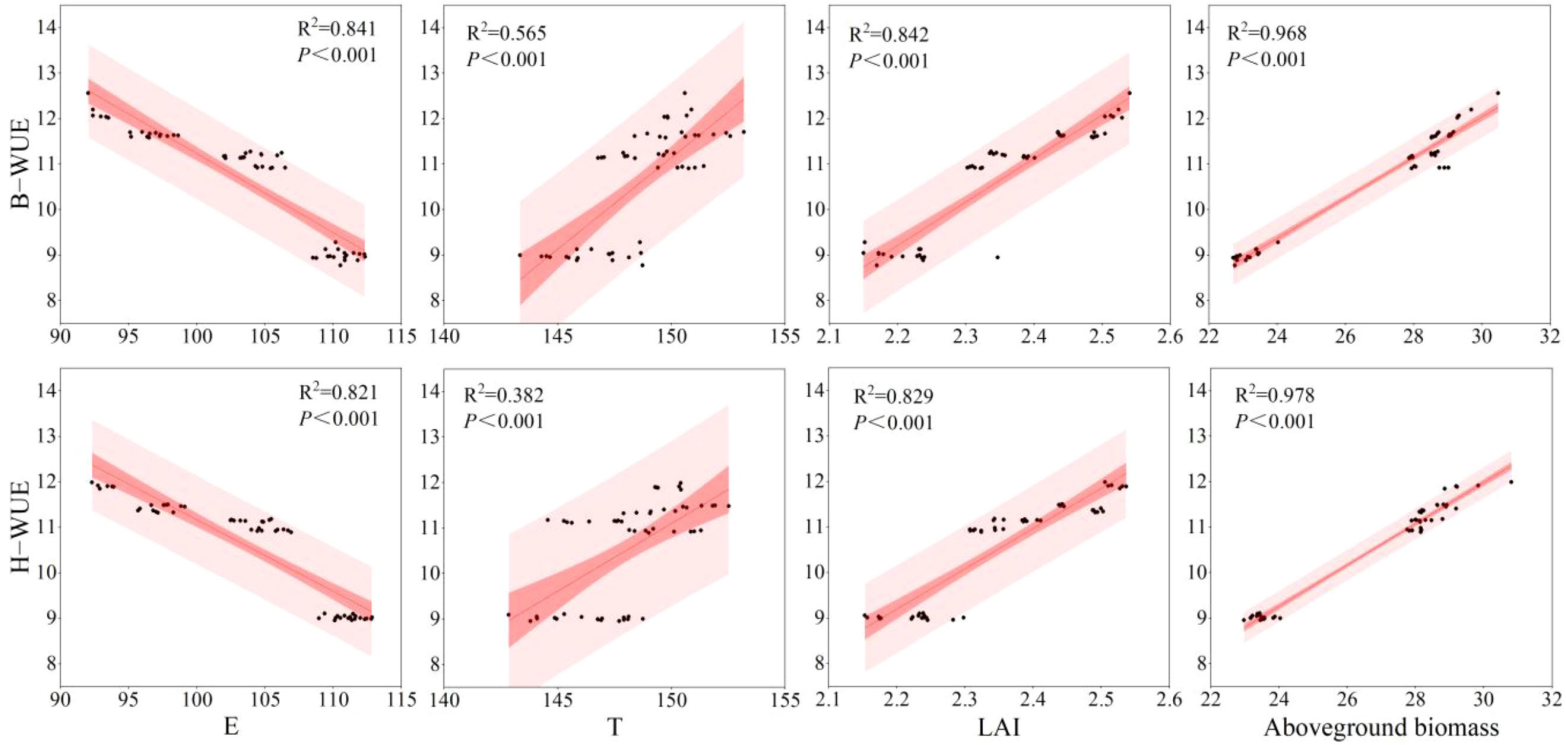
Correlation between water use efficiency (WUE) and various indicators. The dark band represents the 95% confidence interval, while the light band indicates the 95% prediction interval.

Linear functions were used to fit the relationships between each factor and WUE (**Figure 8**). The results indicated that for the two varieties B and H, WUE was significantly and negatively correlated with E under all treatments, with the regression explaining 82.1%–84.1% of the variation in WUE. Meanwhile, T, LAI and aboveground biomass were significantly and positively correlated with WUE, with the regressions explaining 38.2%–56.5%, 82.9%–84.2% and 96.8%–97.8% of the variation in WUE, respectively. It is evident that WUE is influenced by multiple factors.

### 3.8 The key path for enhancing WUE through the interaction between fertilization and density

**Figure 9:**
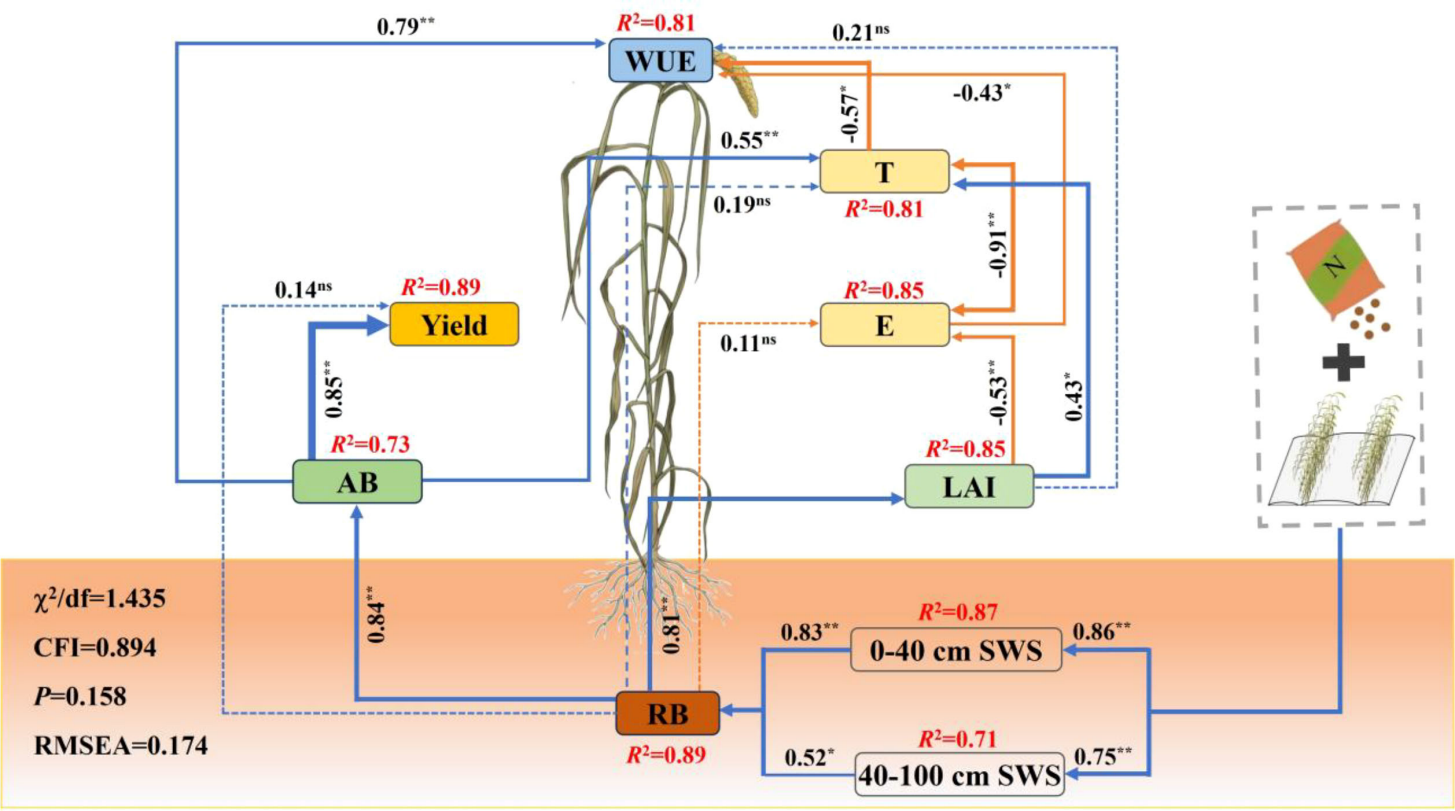
Structural equation modeling (SEM) of the effects of various factors on water use efficiency (WUE). E, T, SWS, AB, RB and LAI represent soil evaporation, transpiration, soil water storage, aboveground biomass, root biomass and leaf area index respectively. The thickness of each arrow and the numbers on the arrows represent the standardized path coefficients and their magnitudes, respectively. Blue lines represent positive correlations, orange lines represent negative correlations, and dashed lines represent no correlations (***P<0.001, **P<0.01, *P<0.05).

SEM was used to analyze the comprehensive response of each factor to WUE (**Figure 9**). The results indicate that the interaction effect between fertilization and density mainly affects the RB (SPC = 0.83**) of foxtail millet by optimizing the SWS of the 0−40cm soil layer, which in turn affects the AB (SPC = 0.84**) and LAI (SPC = 0.81**) of foxtail millet. However, the improvement of WUE is mainly influenced by the positive effect of AB (SPC = 0.79**) and the negative effect of regulating T through LAI (SPC= -0.57*).

## 4 Discussion

### 4.1 Promoting foxtail millet growth and yield enhancement

Crop yield is primarily determined by dry matter accumulation, with optimized planting density serving as a key strategy to enhance population-level biomass production (Zhang et al., 2020; Liu et al., 2023b). Our results demonstrate that the combination of inorganic fertilizer (N2) and moderate planting density (D2, 400,000 plants hm⁻²) achieved optimal growth and yield performance (**Figure 3**). This treatment significantly increased both crop biomass and leaf area index (LAI), consistent with findings by Wu et al. (2024a) in China’s Loess Plateau region. The mechanisms involve: (1) Inorganic nitrogen’s rapid availability promoting leaf expansion and photosynthetic efficiency (Wang et al., 2022); and (2) Moderate density balancing resource utilization (avoiding D1’s underuse and D3’s excessive competition) (Xu et al., 2024). More importantly, their interaction creates a synergistic effect: chemical nitrogen ensures sufficient nutrient supply for individual plant growth, while moderate density optimizes the population structure, allowing plants to more efficiently convert nutrients and moisture into biomass under limited resource conditions. The yield improvement stems from chemical nitrogen meeting crop nutrient demands while optimal density enhances photosynthetic performance. However, some long-term studies suggest organic fertilizers better improve soil structure and nutrient availability−a divergence potentially attributable to our shorter study duration highlighting inorganic nitrogen’s immediate effects, particularly critical in arid/semi-arid regions with limited precipitation windows (Manna et al., 2007).

Notably, the fully mulched double-ridge and furrow sowing system may have amplified these effects by improving soil moisture retention. Practical applications require consideration of local climate, soil texture, and agronomic practices.

### 4.2 Optimizing water consumption characteristics and improving water use efficiency

Water is a critical factor influencing crop growth and yield formation. Both increased planting density and optimized nitrogen management must carefully consider water supply and utilization patterns, especially in dryland cropping systems where efficient water use is fundamental for achieving high and stable yields while improving nutrient utilization efficiency (Wang et al., 2019; Shen et al., 2024; Liu et al., 2025). Our results demonstrate that chemical nitrogen fertilization combined with moderate planting density effectively optimized water consumption patterns in foxtail millet, reducing total seasonal water use while significantly improving water use efficiency (**Figure 3** and **Table 3**). These findings align with Tian et al. (2024), who reported that appropriate chemical nitrogen application promotes soil aggregate formation. The water-holding pores within these aggregates not only retain soil moisture but also preserve water-soluble nutrients, thereby achieving simultaneous improvements in both water and nutrient retention (Bai et al., 2021; Guo et al., 2022). Systematic water monitoring in our study revealed that the chemical nitrogen with moderate density treatment achieved comprehensive improvements in water regulation, manifested through three key increases and one decrease: enhanced soil water storage capacity, increased productive plant transpiration, improved water use efficiency, coupled with reduced unproductive soil evaporation losses. These observations are consistent with Tong et al. (2025), who found that optimized nitrogen application reduces non-productive soil water evaporation while improving crop water productivity.

The underlying mechanisms involve several aspects. First, moderate planting density creates an optimal canopy architecture that improves the utilization efficiency of light and thermal resources. Second, appropriately intensified plant competition under nitrogen-sufficient conditions enhances the crop’s capacity for water and nitrogen acquisition, thereby improving water use efficiency (Fang et al., 2021). Furthermore, chemical nitrogen application facilitates the retention of infiltrated water in upper soil layers, increasing the proportion of water allocated to productive transpiration (Xu et al., 2021). Notably, our study found that the combination of high planting density and organic fertilizer application disrupted the balance between individual plant performance and population productivity, ultimately reducing water use efficiency. This can be attributed to several interconnected factors. Under high-density conditions, the nutrient release from organic fertilizer is too slow to meet the peak nitrogen demand of the dense crop stand, leading to constrained dry matter accumulation during both vegetative and reproductive growth stages (Wu et al., 2024b; Wang et al., 2025). Simultaneously, high planting density intensifies competition for limited soil water, accelerating moisture depletion in the root zone. This water stress, coupled with nutrient limitation, reduces the photoprotective capacity of leaves, accelerates leaf senescence, and ultimately impairs grain filling (Guo et al., 2021). Moreover, the intensified competition under high density increases total seasonal water consumption without a corresponding yield benefit, resulting in a lower proportion of water being used for productive transpiration (Wei et al., 2019; Li et al., 2020).

### 4.3 Relationship between AB, LAI, and WUE

Crop biomass and LAI are key indicators characterizing crop growth and photosynthetic capacity, both closely related to the formation of WUE (Bennetzen et al., 2012; Lin et al., 2024; Sharma et al., 2025; Wang et al., 2023). Results from correlation and SEM in this study confirmed that aboveground biomass (AB) is the core direct factor driving the improvement in WUE, while LAI indirectly influences WUE by regulating the water consumption structure of the population.

AB serves as the fundamental basis for enhancing WUE. In this study, a significant positive correlation was observed between WUE and AB, indicating that efficient biomass production is crucial for achieving high yield per unit of water consumed. The N2D2 treatment (chemical nitrogen fertilizer 120 kg hm^−2^+400, 000 plants hm^−^²) resulted in the highest aboveground biomass, which can be attributed primarily to the rapid nutrient supply from chemical nitrogen fertilizer, which sustained vigorous photosynthesis, and the optimal planting density, which improved population structure by avoiding light resource competition at high density and resource wastage at low density, thereby maximizing the accumulation of photosynthetic products (Guo et al., 2020). SEM analysis further clarified the direct positive path of aboveground biomass on WUE, indicating that under the optimized fertilizer-density regime, crops allocated limited water resources more effectively toward biomass production, establishing a virtuous cycle of “water promoting fertilizer efficiency and fertilizer regulating water use,” ultimately leading to a significant increase in WUE.

LAI indirectly enhances WUE by optimizing the water consumption components (T and ET). The findings of this study revealed a significant positive correlation between LAI and WUE; however, SEM indicated that LAI primarily influenced WUE through its effect on transpiration rather than through a direct path. This elucidates the physio-ecological mechanism by which LAI affects WUE: an appropriate LAI signifies a canopy structure with reasonable closure. Such a structure effectively covers the ground surface, significantly reduces ineffective soil evaporation, and promotes a shift in water consumption toward plant transpiration, thereby optimizing the water consumption structure and increasing the transpiration ratio (Zhang et al., 2016; Wang et al., 2024b). Consequently, the core role of a moderate increase in LAI lies in converting “ineffective” physical evaporation of soil moisture into “effective” physiological transpiration, creating favorable water use conditions for efficient dry matter accumulation.

From an applied perspective, the chemical nitrogen with moderate density regime (120 kg N hm⁻^2^ + 400, 000 plants hm⁻²) developed in this study shows significant practical value for dryland foxtail millet production in the Loess Plateau region. However, special consideration should be given to adjustment of planting density to 350,000 plants hm⁻² during extremely dry years (growing season precipitation<250 mm) to maintain water balance. We acknowledge that the relatively short experimental duration (1–2 growing seasons) may limit comprehensive evaluation of fertilizer-density interaction stability across different climatic conditions, particularly regarding soil water dynamics and crop responses during extreme drought or wet years, which require further investigation. While this study demonstrates that the combination of chemical nitrogen fertilization (N2) and moderate planting density (D2) synergistically enhances yield and water use efficiency in foxtail millet under the fully mulched double-ridge system, it is important to acknowledge its limitations. The experimental design employed three discrete levels of nitrogen (N0, N1, N2) and three discrete density levels (D1, D2, D3). Although this approach successfully identified the optimal treatment combination among those tested, it does not allow for the precise quantification of continuous interaction effects between nitrogen application rate and planting density. Consequently, the exact agronomic optimum within a continuous gradient—such as the specific nitrogen rate that maximizes WUE at a given density, or the density that best balances yield and water use under a given nitrogen supply—remains to be determined. Future research should conduct multi-level gradient experiments, systematically changing nitrogen input and planting density simultaneously on a wider range, and combining the “water−fertilizer−density” synergistic regulation quantitative model for simulation and optimization. Such experiments would not only refine the functional relationships between these two key management factors but also help to identify context-specific recommendations under different soil moisture regimes and climatic conditions, particularly in the face of increasing climate variability in arid and semi-arid regions.

## 5 Conclusions

After the application of chemical nitrogen fertilizer, when the density was moderately increased to 400,000 plants hm⁻², the leaf area index and aboveground biomass of foxtail millet increased simultaneously, and the grain yield increased significantly. Meanwhile, this treatment significantly reduced soil evaporation and increased transpiration, thereby maintaining a relatively low total soil evapotranspiration and significantly enhancing the WUE of foxtail millet. The results of the structural equation model show that the increase in WUE was mainly attributed to the interaction effect of fertilization and density, which promoted root biomass increase by optimizing soil water storage in the 0−40 cm soil layer, thereby influencing aboveground biomass to positively regulate WUE. Therefore, under the condition of chemical nitrogen fertilizer addition, moderate densification (400, 000 plants hm^−2^) can be regarded as a feasible cultivation model for foxtail millet in arid areas to balance high yield and efficient utilization of water resources.

## Data Availability

The original contributions presented in the study are included in the article/supplementary material. Further inquiries can be directed to the corresponding author.
